# Visual target distance, but not visual cursor path length produces shifts in motor behavior: a multisensory integration perspective

**DOI:** 10.3389/fpsyg.2014.00650

**Published:** 2014-06-24

**Authors:** Loes C. J. Van Dam

**Affiliations:** Cognitive Neurosciences, Universität BielefeldBielefeld, Germany

**Keywords:** multisensory integration, visuomotor control, perception of action, action control, multisensory perception

When reproducing a previously performed hand movement, humans do not only take into account the actual hand movement itself, but also visual information about the movement path, for instance via observed cursor movement (Klatzky et al., 2003; Ladwig et al., 2012, 2013). Wendker et al. (2014) studied this phenomenon in a condition in which there where two sources of visual information available. First, participants were presented with two dots on a screen indicating movement starting point and movement target (visual target distance). Second, a visual cursor accompanied participants' hand movement, while direct vision of the hand was prevented. The movement was complete when the cursor reached the target. Interestingly, the visual cursor followed a ∩-shaped path whereas the hand movement itself was physically restricted to be straight. Wendker and colleagues found that, when participants next reproduced the *hand*-movement amplitude in the opposite direction (without any visual information), the path-length along the ∩-shaped cursor path was ignored, but movement amplitudes were still influenced by the previous visual target distance. In other words, Wendker and colleagues show that despite providing largely discrepant information between hand and cursor movement, the visual modality was not ignored as a whole.

Wendker and colleagues interpreted their results based on theories of “Feature Overlap” and “Stimulus-Response Compatibility” (see e.g., Kornblum et al., 1990). In this framework the influence of a particular stimulus on the task depends on its “overlap” with the required response. In correspondence to this approach, Wendker and colleagues considered visual target distance (before movement start) to be overlapping with the hand movement, since they both imply a path along the same horizontal dimension (stimulus-response overlap). Instead, the ∩-shaped cursor movement was considered not to overlap due to its discrepant path.

Here, an additional interpretation from an optimal multisensory integration perspective will be provided. For statistically optimal integration, the different sources of sensory information (here hand movement and visual information) are weighed according to their relative variances (uncertainties): the more variable sensory estimate receiving less weight. The result is a combined estimate that maximizes precision (for a review see e.g., Van Dam et al., forthcoming). From this viewpoint, it becomes interesting to consider the *cursor motion* in its separate X (horizontal) and Y (vertical) components. It has, for instance, been shown that, in terms of their variances, orthogonal directions such as X and Y can be treated more or less independently by the visuomotor system (e.g., Van Beers et al., 1999; Burge et al., 2008). In this light, note that, in Wendker's study, the discrepancy/inaccuracy between hand movement and cursor movement occurred in one direction only: the Y-component of the ∩-shape path. Conversely, cursor movement along the X-dimension was directly linked to hand movement at each point in time. Thus the cursor's X-component fully corresponded with the hand movement in terms of spatiotemporal correlation, an important condition for optimal multisensory integration to occur (e.g., Parise et al., 2012).

This leads to the intriguing question whether cursor movement, in Wendker's study, was indeed ignored as a whole, or whether individual components (in this case the X-component) can still be taken into account for hand-movement perception, based on their respective correspondence with the performed movement. However, it was not the aim of Wendker and colleagues to address this particular question. In their study the cursor always landed on the target, and thus the cursor's X-component and the visual target distance were never disentangled. In other words, from the multisensory integration perspective it is not entirely clear whether the visual information that was being taken into account was the visual target distance (this could for instance indicate movement planning playing a role), or whether it was the X-component of the cursor movement (corresponding to movement execution) that influenced movement perception and reproduction.

To summarize, Wendker and colleagues show that the visual modality was not entirely ignored for movement reproduction, despite providing very discrepant information in terms of cursor movement. This means that the visual information is, in some way, broken down into separate parts. From a “Feature Overlap” perspective, in which only visual target distance can be regarded to overlap with hand movement, it would seem that the cursor movement was completely ignored due to its discrepant ∩-shaped path. However, from an optimal multisensory integration perspective, the study by Wendker and colleagues leads to the interesting question whether a discrepancy in one component of cursor movement naturally leads to the cursor movement being disregarded as a whole, or only in its discrepant counterpart. Answering this question would bring multisensory cue combination research a big step forward, since in most studies no analysis breaking down a signal into its separate components is made. Here, the study by Wendker and colleagues provides an interesting starting point.

## Conflict of interest statement

The author declares that the research was conducted in the absence of any commercial or financial relationships that could be construed as a potential conflict of interest.
